# Клинический случай плюригормональной аденомы гипофиза (СТГ/АКТГ/ТТГ/ФСГ/ЛГ-секретирующая), трудности диагностики

**DOI:** 10.14341/probl13349

**Published:** 2024-09-15

**Authors:** Д. Н. Костылева, П. М. Хандаева, А. М. Лапшина, Е. Г. Пржиялковская, Ж. Е. Белая, А. Ю. Григорьев, Г. А. Мельниченко

**Affiliations:** Национальный медицинский исследовательский центр эндокринологии; Национальный медицинский исследовательский центр эндокринологии; Национальный медицинский исследовательский центр эндокринологии; Национальный медицинский исследовательский центр эндокринологии; Национальный медицинский исследовательский центр эндокринологии; Национальный медицинский исследовательский центр эндокринологии; Национальный медицинский исследовательский центр эндокринологии

**Keywords:** плюригормональная аденома гипофиза, акромегалия, болезнь Иценко-Кушинга, тиреотоксикоз, гиперкортицизм, гонадотропинома, ТТГ-продуцирующая аденома гипофиза

## Abstract

Согласно многочисленным исследованиям, наиболее часто встречающимися образованиями гипофиза являются пролактиномы, достигая 60% от всех клинически значимых образований, за ними следуют гормонально-неактивные аденомы гипофиза, соматотропиномы, кортикотропиномы и тиреотропиномы. Плюригормональные опухоли встречаются менее чем в 1% случаев всех аденом гипофиза. Наиболее распространенная форма аденом со смешанной секрецией в данной популяции пациентов происходит из клеточной линии Pit-1, такие аденомы продуцируют различные комбинации гормонов: гормон роста (ГР), пролактин (ПРЛ), тиреотропный гормон (ТТГ).

В данной статье представлен пациент, имеющий плюригормональную двухкомпонентную макроаденому гипофиза с редким и исключительным сочетанием секретируемых гормонов: соматотропный гормон (СТГ) / адренокортикотропный гормон (АКТГ) / ТТГ / фолликулостимулирующий гормон (ФСГ) / лютеинизирующий гормон (ЛГ) с минимальными неспецифическими клиническими проявлениями в виде сахарного диабета и плохо контролируемой артериальной гипертензии.

## Abstract (en)

According to numerous studies, the most common pituitary tumors are prolactinomas, reaching 60% of all clinically significant adenomas, the next in order are non-functional pituitary adenomas, somatotropinomas, corticotropinomas and thyrotropinomas. Plurigormonal tumors occur in less than 1% of all pituitary adenomas. The most common form of mixed secretion adenoma in this patient population, derived from the Pit-1 cell line, produces various combinations of hormones: growth hormone (GH), prolactin (PRL), thyroid-stimulating hormone (TSH). This article presents a patient with a plurihormonal two-component pituitary macroadenoma with a rare and exceptional combination of secreted hormones — GH / adrenocorticotropic hormone (ACTH) / TSH / follicle-stimulating hormone (FSH) / luteinizing hormone (LH) with minimal nonspecific clinical manifestations such as diabetes mellitus and poorly controlled arterial hypertension.

## АКТУАЛЬНОСТЬ

Опухоли гипофиза составляют до 16% всех первичных новообразований головного мозга и являются третьей по распространенности внутричерепной опухолью после глиомы и менингиомы среди взрослого населения [1].

Эпидемиологические исследования показывают увеличение частоты встречаемости аденом гипофиза от 3,9 до 7,4 случая на 100 000 населения в год и распространенности от 76 до 116 случаев на 100 000 населения среди населения в целом, что может быть обусловлено повышением доступности визуализирующих методов исследования. Аденомы гипофиза все чаще выявляются случайно при выполнении МРТ по несвязанным с аденомой причинам (заболевания ЛОР-органов, неврологические заболевания, черепно-мозговые травмы).

Среди подтипов нейроэндокринных опухолей передней доли гипофиза, по данным многочисленных эпидемиологических исследований, пролактиномы являются наиболее часто диагностируемыми клинически значимыми аденомами гипофиза, составляя 40–60% от общего числа. За ними в порядке убывания следуют нефункционирующие аденомы гипофиза (30%), соматотропиномы (10–15%), кортикотропиномы (4,0–15%), тиреотропиномы (0,3–2%). Менее чем в 1% случаев встречаются плюригормональные аденомы гипофиза, двойные аденомы гипофиза занимают 0,4%–1,3% [1][2][3][4].

Согласно рекомендациям Всемирной организации здравоохранения (ВОЗ), плюригормональными считаются аденомы, секретирующие более одного гормона передней доли гипофиза (за исключением СТГ+ПРЛ, ФСГ+ЛГ секретирующих аденом, которые считаются мономорфными) [5]. Низкая частота выявления плюригормональных аденом гипофиза может быть обусловлена сравнительно недавним внедрением в диагностику иммуногистохимического анализа удаленных опухолей хиазмально-селлярной области с определением специфических факторов транскрипции, гормональной экспрессии, частым отсутствием гиперсекреции гормонов передней доли гипофиза, скудными клиническими проявлениями в случаях, когда гиперсекреция выявлена.

Определение иммунофенотипа продемонстрировало, что плюригормональность часто обнаруживается как в гормонально активных, так и в клинически нефункционирующих аденомах гипофиза. В исследовании Pawlikowski M. et al. показано, что более трети (36,1%) исследованных аденом экспрессируют более одного гормона, что чаще всего не сопровождается гиперсекрецией гормонов гипофиза. По данным исследования Ruoyu Shi et al., функционально-активные плюригормональные аденомы как правило клинически проявляются экспрессией какого-либо одного гормона или вовсе протекают бессимптомно. В настоящее время данные о частоте и клинически-значимых проявлениях плюригормональной аденомы гипофиза все еще немногочисленны и противоречивы [1][5][6].

В соответствии с классификацией эндокринных опухолей, нейроэндокринные опухоли гипофиза (PitNET), ранее известные как аденомы гипофиза, типируются с использованием факторов транскрипции гипофиза (Pit-1, TPIT, SF1, GATA3, ER-альфа), гормонов гипофиза и цитокератинов. В новой классификации применяется корреляция морфологических и иммуногистохимических данных с клиническим течением нейроэндокринных новообразований гипофиза [7].

Наиболее распространенная форма аденом со смешанной секрецией происходит из единой клеточной линии Pit-1, такие аденомы продуцируют различные комбинации ГР, ПРЛ, ТТГ. Однако существуют истинные плюригормональные аденомы с необычными гормональными комбинациями, которые связаны с разными клеточными линиями дифференцировки. Семейство Pit-1 является самым сложным из всех и касается дифференцировки соматотрофных, лактотрофных, маммосоматотрофных и тиреотрофных клеток. Для развития гонадотрофных клеток и соответствующих опухолей необходимы такие факторы транскрипции, как SF-1, ERa, для кортикотрофов, дающих начало также плотногранулированным, редкогранулированым кортикотрофным опухолям, — GATA-2, Lhn4, а также TPIT, NeuroD1. Возможность иммуногистохимического определения специфических факторов транскрипции позволяет уточнить клеточные линии дифференцировки в редких плюригормональных аденомах, как, например, в данном случае [7].

В статье представлено клиническое наблюдение пациента с редкой двухкомпонентной плюригормональной аденомой гипофиза (СТГ/АКТГ/ТТГ/ФСГ/ЛГ-секретирующей) с минимальными клиническими проявлениями. Ранее в литературе не были описаны аденомы с подобным типом секреции гормонов, клинической картиной, особенностями морфологического строения опухоли.

## ОПИСАНИЕ СЛУЧАЯ

Пациент К., 65 лет, обратился на консультацию к неврологу по месту жительства с жалобами на общую слабость, шаткость, неустойчивость походки, отеки, нарушение координации движений, беспокоящие его в течение трех лет. При МРТ головного мозга выявлено образование интраселлярной области с четкими неровными контурами размерами 14х12х17 мм. В связи с выявлением инциденталомы гипофиза был направлен в «НМИЦ Эндокринологии» Министерства здравоохранения России для обследования и определения дальнейшей тактики лечения.

При физикальном обследовании обращают на себя внимание крупные кисти (рис. 1), других изменений внешности по акромегалоидному типу не выявлено. Сам пациент изменений внешности, увеличения размера стоп и кистей не отмечает. Клинических признаков гиперкортицизма нет. Тестикулы сформированы правильно, нормальных размеров, визуальной патологии не выявлено. Масса тела — 77,0 кг, рост — 174 см, индекс массы тела — 25,4 кг/м². Семейный анамнез не отягощен.

**Figure:**
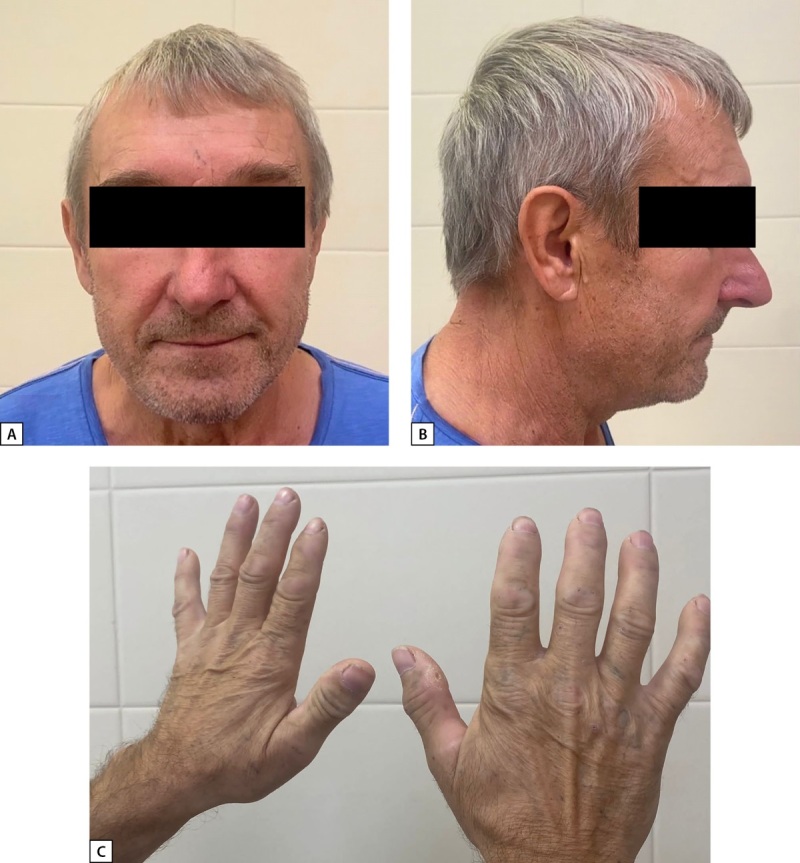
Рисунок 1. Внешний вид пациента.

В ходе клинико-лабораторного обследования выявлена множественная гиперсекреция гормонов гипофиза. Гормональное исследование крови представлено в табл. 1.

**Table:** Таблица 1. Результаты анализов на гормоны до и после оперативного вмешательства

	До аденомэктомии	Через 6 месяцев после аденомэктомии	Единицы измерения	Норма
HbA1c	6,1	5,4	%	4–6
Кортизол свободный в слюне вечером	14,7	13,3	нмоль/л	0,5–9,65
Кортизол вечером	622,8	179,8	нмоль/л	64–327
АКТГ утром	37,15	15,18	пг/мл	7,2–63,3
Кортизол утром	680,4	453,1	нмоль/л	171–536
ТТГ	0,99	0,525	мМе/л	0,25–3,5
свТ4	25,21	12,24	пмоль/л	9–19
свТ3	5,61	2,73	пмоль/л	2,6-5,7
ФСГ	-	4,86	Ед/л	1,6-9,7
ЛГ	-	1,86	ЕД/л	2,5–11
Тестостерон	9,27	13,9	нмоль/л	11–28,2
Пролактин	112	201,2	мЕД/л	60–355
ИФР-1	488,7	192,1	нг/мл	16–245
СТГ	1,89	2,17	нг/мл	0,02–1,23
Паратгормон	24,42	43,7	пг/мл	15–65
Кортизол утром в ходе ночного подавляющего теста с 1 мг дексаметазона	680,4	49,26	нмоль/л	Менее 50
Кортизол свободный суточной мочи	279	451,2	нмоль/сут	100–379
СССГ	72,75	-	нмоль/л	20,6–76,7

В связи с повышением уровня свободного тироксина (свТ4) (25,2 пмоль/л) и референсного уровня ТТГ (0,9 мЕд/л) инициирована терапия аналогами соматостатина. На фоне введения октреотида короткого действия 300 мг/сут., в течение трех суток, нормализовался уровень свободной фракции Т4, значимо снизился уровень ТТГ, что свойственно для ТТГ-продуцирующей аденомы гипофиза. Терапия аналогами соматостатина длительного действия продолжена с целью подготовки пациента к хирургическому лечению для достижения стойкого эутиреоза. Результаты исследования — в табл. 2. Также, учитывая повышение уровня СТГ, инсулиноподобного фактора роста–1 (ИФР-1), проведена проба с нагрузкой глюкозой (СТГ в ходе орального глюкозотолерантного теста), подавления СТГ достигнуто не было, что свидетельствует в пользу активной стадии акромегалии (табл. 3). Вместе с тем отмечалось повышение кортизола сыворотки вечерней крови и слюны, отсутствие подавления кортизола в ходе ночного подавляющего теста с 1 мг дексаметазона на фоне референсных значений свободного кортизола суточной мочи.

**Table:** Таблица 2. Результаты анализов на тиреоидные гормоны до и после пробы с аналогами соматостатина

	До проведения пробы	Через трое суток от начала пробы	Единицы измерения	Норма
ТТГ	0,99	0,138<	мМе/л	0,25–3,5
свТ4	25,21	20,7	пмоль/л	9–19
свТ3	5,61	-	пмоль/л	2,6–5,7

**Table:** Таблица 3. Результаты анализов СТГ в ходе орального глюкозотолерантного теста

	До хирургического лечения	После хирургического лечения	Единицы измерения
СТГ, на 0 минуте	3,07	0,29	нг/мл
СТГ, 30 минут после пробы	1,74	0,33	нг/мл
СТГ, 60 минут после пробы	3,65	0,68	нг/мл
СТГ, 90 минут после пробы	2,17	1,09	нг/мл
СТГ, 120 минут после пробы	1,99	0,60	нг/мл

Таким образом, верифицирована опухоль гипофиза размерами 13х20,5х18 мм с лабораторно плюригормональной секрецией (СТГ/АКТГ/ТТГ-секретирующая) с минимальными клиническими проявлениями: сахарный диабет (СД), артериальная гипертнезия (АГ). При офтальмологическом осмотре данных за хиазмальный синдром не получено.

С учетом наличия лабораторных признаков гормональной гиперсекреции, в стационаре проведено обследование на предмет наличия осложнений основного заболевания. При оценке углеводного обмена подтвержден СД, выявленный в 2021 г. На фоне получаемой сахароснижающей терапии (дапаглифлозин 10 мг), несмотря на целевой показатель гликированного гемоглобина (HbA1c) — 6,1%, по данным гликемического профиля, за период госпитализации наблюдалась вариабельность показателей гликемии от 4,9 до 15,2 ммоль/л. В связи с выявленной кетонурией на фоне приема дапаглифлозина произведена коррекция сахароснижающей терапии: переведен на инсулинотерапию в базис-болюсном режиме. На фоне скорректированной терапии достигнуто снижение вариабельности гликемии в течение суток, тенденция показателей гликемии к целевым (максимальное повышение гликемии до 8,5 ммоль/л). Подтверждена АГ III степени (c достижением оптимального контроля АД на фоне плюрикомпонентной медикаментозной терапии (БРА, БМКК и диуретик)), 2 степени с высоким риском неблагоприятных кардиоваскулярных событий. Также, по данным УЗИ щитовидной железы, определялись эхографические признаки двустороннего многоузлового зоба (EU-TIRADS2) с фокальными изменениями в обеих долях на фоне аутоиммунного поражения щитовидной железы. По результатам рентгеновской денситометрии данных за снижение минеральной плотности костей получено не было.

С учетом наличия плюригормональной аденомы гипофиза рекомендована трансназальная трансфеноидальная аденомэктомия.

16 июня 2022 г. выполнена трансназальная транссфеноидальная аденомэктомия. Во время нейрохирургического вмешательства и ревизии турецкого седла обнаружена опухоль разной плотности с петрификатами. В передней части турецкого седла опухоль умеренно плотной консистенции (не аспирировалась стандартным отсосом) имела петрифицированные фрагменты. Образование удалено при помощи опухолевых кусачек и кюреток. Образцы удаленной опухоли были промаркированы как образец №1. Затем удалена вторая часть опухоли, которая была серого цвета, мягкой консистенции и располагалась в задней части седла — образец №2.

При патоморфологическом исследовании материал представлен опухолью гипофиза различного строения. Образцы ткани №1 содержали опухоль гипофиза солидного строения из клеток с оксифильной и хромофобной цитоплазмой (митозы не определялись) с обширными участками обызвествления. Образцы ткани №2 содержали опухоль преимущественно из хромофобных клеток, формирующих многочисленные периваскулярные псевдорозетки, митозы не обнаружены. По результатам иммуногистохимического исследования в образце №1 обнаружены диффузно расположенные клетки опухоли, позитивные к АКТГ, гормону роста и ТТГ, также в ядрах клеток имелась экспрессия соответствующих транскрипционных факторов (TPIT, Pit-1). Экспрессия пролактина, ЛГ и ФСГ, а также эстрогеновых рецепторов альфа (ERα) и SF1 не определялась. В образце №2 выявлен иной иммунофенотип: клетки опухоли были позитивны к ЛГ, ФСГ и SF1 при отсутствии экспрессии АКТГ, гормона роста, пролактина, ТТГ и соответствующих указанной клеточной дифференцировки транскрипционных факторов (TPIT, Pit-1, ERα). Обращает на себя внимание несколько отличающийся уровень пролиферативной активности по данным индекса метки Ki-67: в образце №1 индекс метки составил 1,8%, в образце №2 — 2,8%. Опухоль из двух образцов была иммунопозитивна к низкомолекулярному цитокератину (СAM 5.2). Таким образом, морфологическая картина была интерпретирована как истинная плюригормональная аденома гипофиза, один из компонентов которой представлен кортикотрофными, тирео- и соматотрофными клетками с меньшей пролиферативной активностью. Второй компонент — гонадотрофными клетками с более высокой пролиферативной активностью (рис. 2).

**Figure:**
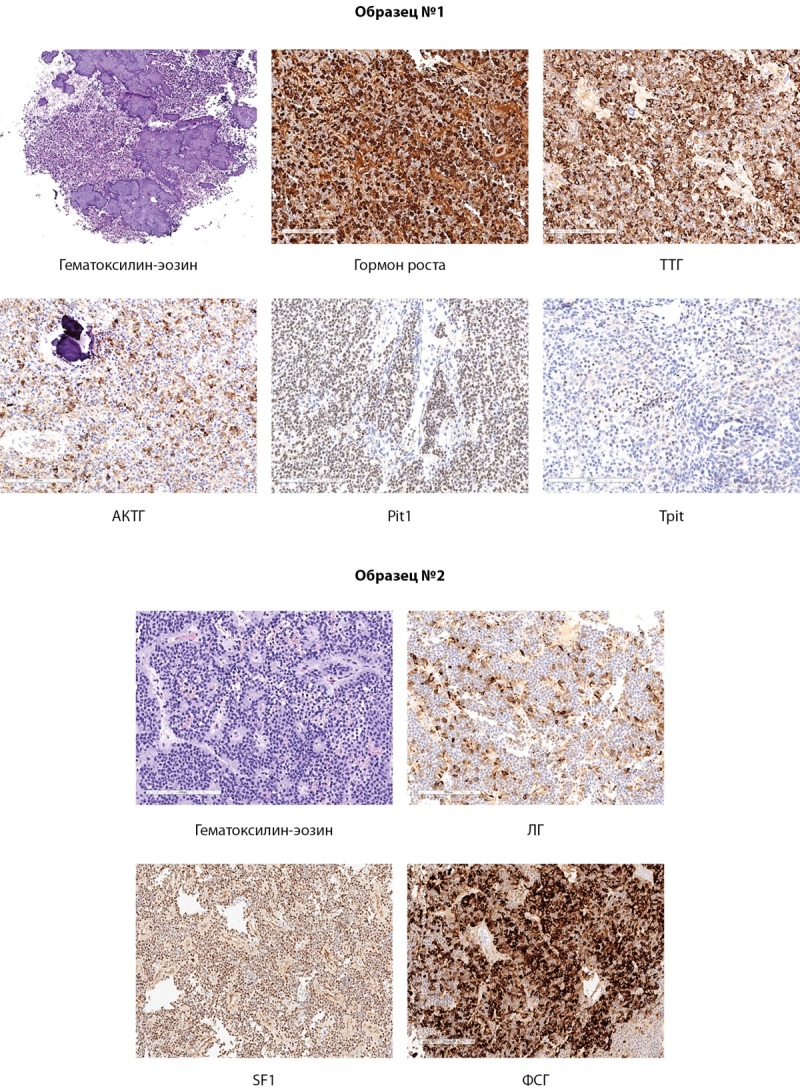
Рисунок 2. Микроскопическое строение опухоли из разных образцов удаленной ткани.

Послеоперационный период протекал без особенностей, развития осложнений, пациент отмечал улучшение общего состояния, регресс общей слабости, шаткости при походке, отечности лица, нижних конечностей. По результатам лабораторного контроля получены дискордантные данные в отношении персистенции эндогенного гиперкортицизма: отмечается повышение кортизола в вечерней слюне (12,9 нмоль/л (0,5–9,65) при нормализации его уровня в суточной моче (366 нмоль/сут (100–379)). Также наблюдалось подавление СТГ в ходе ОГТТ до 0,2 нг/мл (табл. 3), снижение уровня ТТГ — 0,005 мМЕ/л (0,25–3,5) при нормальных показателях свТ3 и свТ4, что подтверждает достижение послеоперационной ремиссии акромегалии и центрального тиреотоксикоза.

После оперативного лечения, в связи с нормализацией АД, отменена антигипертензивная терапия, учитывая целевые значения гликемии — отменена инсулинотерапия, пациент переведен на пероральную сахароснижающую терапию: метформин 500 мг 2 р/день, вилдаглиптин 50 мг 2 р/день, на фоне чего отмечается целевой гликемический профиль.

В ходе повторной госпитализации в октябре 2022 г., через 6 месяцев после нейрохирургического лечения, по данным клинико-лабораторного исследования, получены данные, свидетельствующие о нормализации секреции гормона роста и ТТГ с сохранением дискордантных показателей в отношении центрального гиперкортицизма: нарушен суточный ритм секреции АКТГ, отмечено повышение кортизола в вечерней слюне и суточной моче, при нормальном показателе кортизола вечерней крови, положительном ночном подавляющем тесте с 1 мг дексаметазона. Гормональное исследование крови представлено в табл. 2. Выполнена МРТ головного мозга с контрастным усилением, по результатам которой МР-картина соответствует послеоперационным кистозно-фиброзным изменениям (зона измененного сигнала в центральных и левых отделах аденогипофиза размерами 16х13х9 мм). Пациент консультирован нейрохирургом, показания к повторному хирургическому лечению в настоящее время отсутствуют, показано динамическое наблюдение.

## ОБСУЖДЕНИЕ

Диагностика плюригормональных аденом гипофиза на начальном этапе затруднительна. Основная сложность своевременной диагностики может заключаться в отсутствии яркой клинической картины, несмотря на имеющуюся гиперсекрецию гормонов гипофиза [6]. Например, в описанном Felix I. et al. клиническом случае у пациента с двойной макроаденомой гипофиза, положительной на ГР, ТТГ и а-субъединицу, по результатам иммуногистохимического исследования, с повышенным ТТГ при референсных значениях свободных Т3 и Т4 в сыворотке крови, — не отмечалось признаков центрального тиреотоксикоза, а проявления акромегалии были не ярко выражены клинически и биохимически [8]. В проведенном Wang M. et al. сравнительном исследовании, в которое было включено 279 пациентов с соматомаммотропиномами либо чистыми СТГ, ПРЛ продуцирующими аденомами, отмечено, что у пациентов со смешанными аденомами клинические проявления акромегалии были более скудными, также не были выражены сердечно-сосудистые и обменные нарушения [9]. В другом недавно опубликованном систематическом обзоре литературы был проанализирован 21 случай плюригормональных аденом гипофиза со смешанной секрецией АКТГ и ГР, из них в 2 случаях были выражены признаки гиперкортицизма, в 5 — одновременно акромегалии и гиперкортицизма, в 11 — признаки акромегалии. Интересно, что в шести случаях, помимо АКТГ и ГР, наблюдалась секреция ПРЛ [10].

При плюригормональных аденомах гипофиза клиническая картина, обусловленная гиперпродукцией АКТГ, встречается редко, с частотой 3,6% [11]. Это позволяет предположить, что гормоны, выделяемые опухолью, не всегда являются биологически активными или утрачивают свою активность при попадании в кровоток. Интересной также является возможность трансформации гормонально-секретирующей аденомы гипофиза из одного типа секреции в другой, что чаще наблюдается при синдромах множественных эндокринных неоплазий [12].

Определение биологической активности гормонов, выделяемых опухолями данного типа, ранее не исследовалось и может являться перспективным направлением лабораторной диагностики.

Нередко плюригормональные аденомы дебютируют с СД или АГ, что находит подтверждение в описанном клиническом случае и в ряде других [13]. Стоит отметить, что плюригормональные аденомы склонны к агрессивному течению, особенно в случае косекреции АКТГ ввиду высокой частоты рецидивов [5][14]. В представленном клиническом случае у пациента сохраняется повышение кортизола в вечерней слюне и суточной моче при нормальном показателе кортизола вечерней крови, положительном ночном подавляющем тесте с 1 мг дексаметазона, что может свидетельствовать о персистенции эндогенного гиперкортицизма. Однако ввиду отсутствия данных о наличии остаточной ткани аденомы по данным МРТ и клинических проявлениях заболевания, пациенту рекомендован динамический контроль.

При изучении литературы также отмечается, что у пациентов со смешанной секрецией гормонов разных клеточных линий дифференцировки, например АКТГ, ТТГ, СТГ и ФСГ/ЛГ, в большинстве случаев речь идет о аденомах гипофиза с секрецией двух гормонов [6][15].

Стоит обратить внимание, что в рассматриваемом клиническом случае в ходе хирургического лечения визуально отмечены два компонента с разными морфологическими и иммуногистохимическими характеристиками, что, вполне вероятно, может говорить о наличии двойной аденомы гипофиза, где одна часть опухоли продуцирует АКТГ, ТТГ и ГР, а вторая — ФСГ и ЛГ; ранее в литературе подобные аденомы гипофиза не были описаны.

Данный клинический случай демонстрирует важность проведения иммуногистохимического исследования послеоперационного материала, что позволяет уточнить характер секреции опухоли, ее гетерогенность, тем самым верифицировать диагноз и определить дальнейшую тактику лечения и динамического наблюдения.

## ЗАКЛЮЧЕНИЕ

Впервые нами описан клинический случай пациента с макроаденомой гипофиза с исключительным сочетанием гормональной продукции (СТГ, АКТГ, ТТГ, ФСГ и ЛГ) при отсутствии тяжелых проявлений акромегалии, болезни Иценко-Кушинга, центрального гипертиреоза.

Описанное наблюдение иллюстрирует необходимость внедрения в рутинную практику иммуногистохимического исследования с определением не только экспрессии всех рекомендованных ВОЗ гормонов, но и транскрипционных факторов, что может увеличить частоту выявления плюригормональных аденом гипофиза, а отсутствие корреляции между экспрессией гормонов гипофиза с их сывороточной концентрацией и выраженностью клинических синдромов может стать стимулом для разработки новых способов диагностики.

## ДОПОЛНИТЕЛЬНАЯ ИНФОРМАЦИЯ

Источники финансирования. Исследование проведено при поддержке Российского научного фонда (грант РНФ 19-15-00398).

Конфликт интересов. Авторы декларируют отсутствие явных и потенциальных конфликтов интересов, связанных с содержанием настоящей статьи.

Участие авторов. Все авторы одобрили финальную версию статьи перед публикацией, выразили согласие нести ответственность за все аспекты работы, подразумевающую надлежащее изучение и решение вопросов, связанных с точностью или добросовестностью любой части работы.

Согласие пациента. Пациент добровольно подписал информированное согласие на публикацию персональной медицинской информации в обезличенной форме.
